# Navigating HOPE (Hypermobile Online Pain managemEnt): Perspectives and Experiences From People With Hypermobile Ehlers–Danlos Syndrome or Hypermobility Spectrum Disorder on a Condition‐Specific Online Pain Management Programme

**DOI:** 10.1111/hex.70186

**Published:** 2025-02-17

**Authors:** Min Tze Chew, Emre Ilhan, Sarah Dennis, Leslie L. Nicholson, Sarah Kobayashi, Cliffton Chan

**Affiliations:** ^1^ Department of Health Sciences, Faculty of Medicine, Health and Human Sciences Macquarie University Sydney New South Wales Australia; ^2^ School of Health Sciences, Faculty of Medicine and Health, The Susan Wakil Health Building University of Sydney Sydney New South Wales Australia; ^3^ Ingham Institute of Applied Medical Research Liverpool New South Wales Australia; ^4^ Kolling Institute, Faculty of Medicine and Health University of Sydney Sydney New South Wales Australia

## Abstract

**Introduction:**

The Hypermobile Online Pain managemEnt (HOPE) programme is a stakeholder informed intervention adopting the biopsychosocial pain approach, specifically for people with hypermobile Ehlers–Danlos Syndrome (hEDS) and hypermobility spectrum disorder (HSD) experiencing pain. The programme topics included were based on a modified Delphi of a large sample of stakeholders: people with hEDS/HSD and healthcare practitioners who are experienced with managing these conditions. Programme feasibility, acceptability and appropriateness were previously evaluated quantitatively in a pilot randomised controlled trial, but the in‐depth experiences and perceptions of participants who engaged with the programme is unknown.

**Methods:**

Qualitative study. 1:1, semi‐structured online interviews of participants who took part in the HOPE programme. Data was analysed using an inductive thematic analysis approach as described by Braun and Clark.

**Results:**

Twelve participants were interviewed; 91% were female, mean age 38.1 (SD 9.1). Four themes emerged: (1) The biopsychosocial approach to understanding pain used in the HOPE programme was acceptable and appropriate, (2) benefits of the programme were stronger for those who were newer on their hEDS/HSD journey, (3) self‐guided reflections included in the programme required headspace and personal meaning and (4) participants desired more adaptable content and programme functionality. Additionally, participants gave suggestions on how to improve the content, adherence and engagement to the programme.

**Conclusion:**

The HOPE programme was considered feasible, acceptable and appropriate for people with hEDS/HSD. The four themes and suggestions from our study findings will be used to refine subsequent versions and large‐scale trials of the HOPE programme, as well as provide translatable insights for other online interventions for hEDS/HSD or similar complex, chronic multisystemic conditions.

**Patient or Public Contribution:**

A large community of hEDS/HSD patients' and healthcare providers' input were obtained from a two‐staged online Delphi from a prior study. This approach was preferred to capture the greatest amount of feedback from a diverse international voice. Via the Delphi study, they provided suggestions for content topics and consensus on what they felt were important to include in a hEDS/HSD specific online pain management programme, as well as programme parameters (e.g., duration and frequency of programme; healthcare provider telehealth component; types of learning activities).

## 1 Introduction

Hypermobile Ehlers–Danlos syndrome (hEDS) and hypermobility spectrum disorder (HSD) are connective tissue disorders displaying multisystemic signs and symptoms including widespread pain, neuromusculoskeletal impairments, urogenital symptoms, anxiety, depression, cardiac dysfunction, gastrointestinal dysfunction and fatigue [1]. Up to 98% of people with hEDS/HSD experience pain [2, 3], and those with severe pain experience greater disability [4]. Pain is one of the primary reasons for seeking health care [5], and multidisciplinary management encompassing the biopsychosocial approach to pain is current best practice for this population [6, 7, 8].

In‐person multidisciplinary treatment programmes for the adult hEDS/HSD population significantly improve pain‐related measures, function, emotional status, strength and endurance [9, 10, 11, 12, 13]. However, they require considerable resources and this often limits their delivery to large hospital‐based or tertiary‐based settings [14], which may not be easily accessible to the wider population.

Online pain interventions may enable specialised, high‐value care from anywhere and at any time [15, 16]. People with hEDS/HSD often have unpredictable symptoms; their mobility and activity tolerance change markedly day‐to‐day [17]. This can result in them missing out on important planned medical appointments. Online asynchronous interventions may enable individuals to access pain management in their own time and place. For example, the online Pain Course, a generic pain programme based on Cognitive Behavioural Therapy principals, has shown feasibility, acceptability and long‐term effectiveness in pain‐related disability, emotional states [18, 19, 20], and potential for cost savings [21]. However, non‐condition‐specific programmes for conditions where complex biology plays a significant role in one's pain experience, may not be sufficient for people with hEDS/HSD [22, 23, 24]. Indeed, when a group of hEDS/HSD participants (*n* = 396) was surveyed, 81% indicated that a hEDS/HSD‐specific online pain management programme was important or very important [25]. Therefore, a dedicated hEDS/HSD online pain programme may be appropriate and acceptable.

The Hypermobile Online Pain managemEnt (HOPE) programme was developed using a three‐staged process including stakeholder input [25]. Pilot testing determined its feasibility, acceptability and appropriateness [26]. What remained unknown is ‘why’ and ‘how’ the programme impacts people [27]. Furthermore, current best‐practice guidelines using the biopsychosocial model to address pain are recommended by the medical profession [6, 7], but the perspectives of people with hEDS/HSD who undergo these treatment paradigms are unknown. The main aim of this qualitative study is to deeply explore participant perspectives on the feasibility, acceptability and appropriateness of the HOPE programme and use these findings to guide the design and implementation of future interventional studies or programmes for hEDS/HSD cohorts.

## 2 Methods

### 2.1 Participant Recruitment

This study was approved by the Macquarie University Human Research Ethics committee (520231630154261). Participants were recruited from the pool of intervention participants in the quantitative study [26]. Purposeful sampling [28] was used to ensure representativeness across gender identity, programme completion rate (completers defined as completing ≥ 75% of the programme, non‐completers as completing ≤ 50%), education level (university, certificate or high school), and Patient Global Impression of Change scores (improved, no change and worsened).

### 2.2 Author Reflexivity

All authors contributed experiential knowledge to study design and interpretation of data, including experience of generalised joint hypermobility (GJH) (MTC and CC), providing physiotherapy care to people with hEDS/HSD (MTC and CC), EDS research (MTC, LN and CC), pain research (EI and SK), health services research and qualitative methods (SD).

The primary author (MTC), who was the primary data analyst, utilised a contextualist approach when interpreting data from her and participants' perspectives, opinions, context and experiences. Her lived experiences of GJH were made known to all participants and it was reinforced that their experiences were unique to lend voice to their own lived experiences.

### 2.3 Data Collection and Analysis

The primary author conducted all interviews, and an experienced qualitative researcher (SD) supervised the first three interviews. Interviews were conducted virtually using Microsoft Teams (version 24295.606.3238.6194), with audio and/or video recordings. Informed written and verbal consent was sought from participants before their interview. Participants were reimbursed for their time. The semi‐structured interview included questions about the feasibility, acceptability and appropriateness of the HOPE programme, and suggestions for improvements (Supporting Information: A).

Audio files were transcribed using Microsoft Teams and converted into Microsoft Word (version 16.90.2) files. The primary author reviewed transcripts against the audio and/or video for accuracy. The checked transcripts were sent to each participant for review with a 5‐day response period. Only one participant provided minor spelling and grammar changes.

Data analysis was performed using inductive thematic analysis as described by Braun and Clark [29]. Initially, the primary author familiarised herself with each data set, making notes and recording features that were relevant to the research question. After the first and every subsequent third participant transcript, the analysis was discussed with the team (EI, SD and CC). The second step involved generating initial minor codes recorded using NVivo (version 14.23.0). From these, initial major codes and themes were generated by organising similar codes together, ending with a set of candidate themes for the third step. In the fourth step, candidate themes were reviewed and developed further by exploring if they were coherent with all coded data within and across all datasets. The fifth step involved refining candidate themes, defining and renaming them where appropriate to ensure that they addressed the research question. The fourth and fifth steps were done collaboratively and iteratively with the research team. The wider group discussed, revised and refined each theme, with discussions recorded as a mind map for record‐keeping. In the final step, the primary author wrote the analysis presented in the results section.

Interview questions asked were based on the three domains of feasibility, acceptability and appropriateness but participants were allowed to deviate to provide broader perspectives. Data analysis was performed inductively where all codes were given equal opportunity to arise, even if they did not fall within the three domains. Therefore, the results section is presented in a narrative format where each theme encompassed the three domains to varying degrees and included additional themes beyond them.

## 3 Results

Of the 36 participants in the intervention group, all 21 participants who completed their post‐intervention outcomes were invited to take part in the interview; three declined and six did not respond. Twelve consented and were interviewed between April and August 2024. Interviews lasted 25–55 min, with breaks as required. Demographically, 91% were female, mean age 38.1 (SD 9.1), 75% had a university degree and 58% currently or previously worked in health care (Table 1).

**Table 1:** Participant demographics.

ID	Age range	Completion status	Gender	Highest level of education	Field of work	Patient global impression of change (PGIC)
A	20–29	≥ 75%	F	University	Healthcare student	No change
B	30–39	≥ 75%	F	University	Unknown	Minimally improved
C	30–39	≥ 75%	F	University	Education	Much improved
D	40–49	≥ 75%	M	High‐school	Pensioner (previously worked in health care)	No change
E	50–59	≥ 75%	F	University	Health care	No change
F	30–39	≥ 75%	F	University	Visual communications (previously in fitness industry)	No change
G	30–39	≥ 75%	F	University	Health care	No change
H	40–49	≤ 50%	F	University	Information services and management	Minimally worse
I	30–39	≥ 75%	F	Certificate	Health care and business	No change
J	30–39	≥ 75%	F	University	Health care	No change
K	50–59	≥ 75%	F	Certificate	Complementary and alternative health care	No change
L	30–39	> 50%, < 75%	F	University	Archaeology	Minimally improved

We made follow‐up phone calls and sent emails to all programme non‐starters (*n* = 7) to find out their reasons for not starting the programme. Only two participants responded, citing medical issues as their reason.

**Figure 1:**
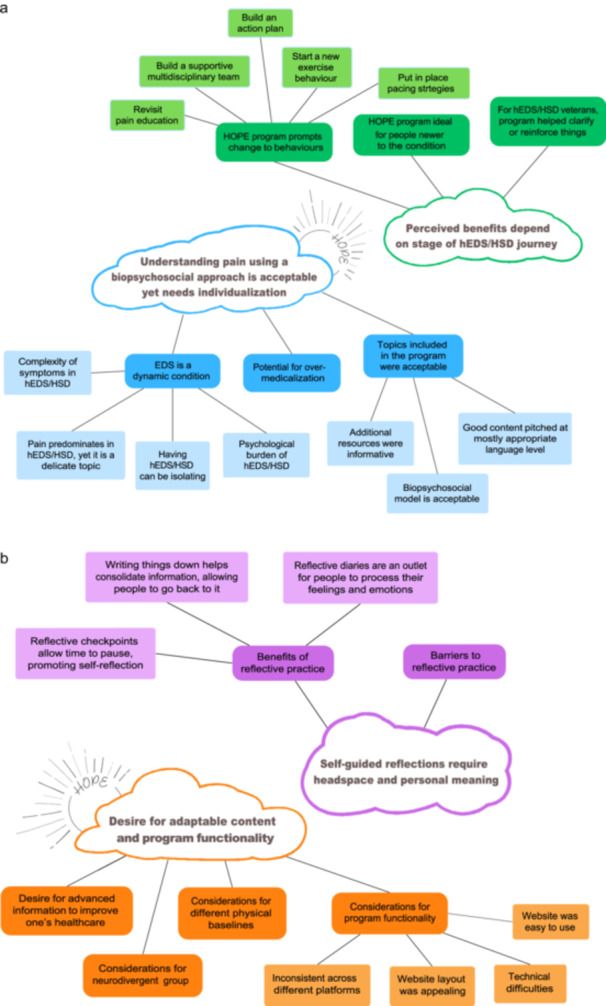
(a) Themes 1 and 2 with their respective major and minor codes. (b) Themes 3 and 4 with their respective major and minor codes.

Data saturation was reached with the 12 interviews. Four major themes were identified. Figure 1a,b depicts the major and minor codes that were grouped to form their respective themes. There were two standalone themes, presented as ‘incidental themes’. These themes do not address our aims but were consistently conveyed across interviews and hence warrant inclusion. Lastly, ‘suggestions for improvements’ are presented in the closing section of the results.

Theme 1 Biopsychosocial approach to understanding pain is acceptable yet needs individualisation.

The HOPE programme content was created based on the biopsychosocial approach to pain and most participants felt it was acceptable and pitched at an appropriate level.

‘I really liked how it started off at the background, explaining how pain works, how your body works … And then I liked how it also explained […] your feelings and things and your lifestyle’ (E).

‘But particularly I found the brain processing of pain space quite interesting and new. It's been something that I've then sort of discussed with support and loved ones […] to try […] not to, I guess, discourage feeling what you're feeling, but also, are there things that I can sort of do to try and refocus my mind and perhaps not just sit in that space of you can't get ahead of feeling the pain…’ (L).

Participants appreciated additional resources which included links to further readings and journal articles that provided scientific evidence to aid their understanding.

‘Having the ability to go out and read […] more scientifically, I found that was really supportive […] when you're working with your physio and you know you're sort of writing things on whiteboard and you're talking about YouTube videos and it can seem a little social media‐rised. And as a librarian… I want my triple check peer reviewed.’ (H)

While most found the content acceptable, there were concerns about some topics having potential for over‐medicalization with content detailing signs/symptoms and comorbidities of hEDS/HSD.

‘I found that perhaps some of the resources were a bit suggestive of, vulnerable people might think, oh yes, I might have that too, when previously it's not been a consideration…’ (J)

One participant approached the pain science content cautiously, particularly due to the potential for triggering those who were medically traumatised.

‘I understand that is a lot of the neurological links and things, but I think sometimes that can be a trigger to someone. You're telling me it's in my head again because I think going through that long process of getting a diagnosis, how many people have been (told), it's all in your head […] And I think that needs a lot more delicate interpretation.’ (K)

Another related point that emerged was that hEDS/HSD are dynamic conditions, and providing individualised care is important. Each person's experience is different due to the variability in multi‐systemic involvement.

‘…that's usually the case for someone with hEDS. You've got lots of things going on at the same time’. (F)

‘I don't think I've ever been more than three months without seeing a physio.’ (A)

Among our participants, there were comorbidities including fibromyalgia, dysautonomia, bowel bleeding and neck issues. One participant described their ‘*entire childhood was at the physio’ (K)*, for various issues, which was echoed by another who had a ‘*mix of symptoms my whole life, we've always just treated every symptom*’ *(C)*. One participant reported symptom fluctuations related to the weather. With such varied experiences, pain management needs to be individually tailored.

The importance of adopting the entire biopsychosocial approach for hEDS/HSD is supported by our participants' experience of psychological and social burdens. Participants expressed various psychological difficulties, such as ‘*struggling mentally*’ *(E)*, ‘*working on the level of fear*’ *(H)*, ‘*stressed*’ *(B)* and ‘*panicked*’ *(B, D)*, ‘*exhaustion… hopelessness*’ *(D)* and ‘*an emotional roller coaster*’ *(C)*. Socially, some participants expressed loneliness. One participant mentioned isolation due to poor healthcare experiences, another verbalised that many people with hEDS/HSD ‘*really struggle with the kind of loneliness that comes from being in […] weird dynamics with their health professionals*’ and ‘*isolation is a big element*’ *(G)*. Several participants found difficulty with online social groups. While intended to provide support, one participant said, ‘*sometimes it's just people posting all their injuries and I don't want to see this*’ *(L)* and another commented that ‘*it only brings me down…*’ *(D)*.

Theme 2 Perceived benefits of the HOPE programme are dependent on stage of hEDS/HSD journey.

Many participants reported spending years researching in attempt to understand their condition because the journey to receiving a hEDS/HSD diagnosis and finding targeted medical care can be long and challenging. Having developed hEDS/HSD literacy, these participants, figuratively referred by us as ‘hEDS/HSD veterans’, felt that the HOPE programme did not provide new information.

‘Originally it was appealing because I thought, this might be teaching me something that I didn't know, or helping the pain. I don't think it helps the pain, but […] I think it could be because […] most of it wasn't new.’ (F)

‘Well, I've done a lot of research myself over the last few years so in that regard […] I wouldn't have said there was any new information there for me.’ (D)

These ‘veterans’ felt the programme would be more beneficial for people early in their journey. Indeed, participants in the early stages of their diagnosis reported stronger perceived benefits from the HOPE programme.

‘For a person who doesn't have the knowledge that I do, I think it's fantastic. I think it would be very helpful”; “that's really good if I had this about maybe 12 years ago’ (E)

‘I think the people who are just recently diagnosed or who aren't super health literate, it does benefit them.’ (G)

‘For someone who was newly diagnosed and very overwhelmed […] I was despairing of my diagnosis. The content was an absolute godsend…’. (C)

Some ‘hEDS/HSD veterans’, however, reported that the programme prompted them to change certain behaviours. One participant revised previous pain education. Another started a new type of exercise: ‘*I like the tips and exercise so I actually ordered a Pilates reformer… It did encourage me […] to keep moving…*’ *(E)*. Two participants were prompted to develop their pain action plan.

‘Even not having it written down would be really helpful. But even just thinking through it myself, […] the first step would be this, and then this and then this, I realised, was actually very helpful’ (F)

‘I need to have a step‐by‐step plan so that my husband actually knows what to do… he doesn't know what to do half the time. So […] I do need to write that for my family.’ (K)

The programme helped participants consider who to include in their multidisciplinary team such as a pain specialist, or a chiropractor, exercise physiologist and psychologist.

Some participants were reminded about the importance of pacing.

‘Staying on top of things a bit better… I'm less crash and burn‐y. Boom, busty.’ (A)

Another participant described struggling with pain due to hyper‐focusing while doing computer work. The programme reminded her to incorporate pacing strategies such as setting a different task after a certain number of hours on the computer.

Theme 3 Desire for adaptable content and programme functionality.

Many ‘hEDS/HSD veterans’ desired adaptability of the programme content with new or advanced information that could improve their health care. Since more than half of our participants worked or studied in healthcare fields, their health literacy appeared relatively high.

Several participants reported being neurodivergent and pointed out helpful aspects of the programme, such as bite‐sized chunks of content and multiple points at which they could choose to pause and resume. Conversely, some participants indicated features that made it hard to concentrate, including moving animations placed next to text. One participant also commented that the programme was somewhat serious, ‘*structured like a course, it probably triggered a lot of their, I'm at school type thing*’ *(G)* that might have been off‐putting.

The programme included a section on cognitive behavioural therapy (CBT). Two participants who underwent this approach in the past found that CBT did not help them, and they expressed difficulty engaging in that section because it triggered negative memories from undergoing CBT.

Another consideration raised was that the programme insufficiently catered for people with different physical baselines. For example, the programme included pictures and videos of sitting exercises. One participant with dysautonomia and chronic fatigue could only exercise lying down. Another participant stated that age may affect exercise suitability.

‘Someone who's 20, […], 30, […] 40, […] 50 and older, they're all going to have very different capacities and that's where they'll look at an exercise and just go, I'm not even going there… You have to be able to come back and work at where's their level when they're dealing with so much.’ (K)

In terms of programme functionality, participants felt the website was easy to use (*A*, *B*, *C*, *E*, *F*, *G*, *I* and *K*) and had an appealing layout (*A*, *E* and *I*). While we pre‐instructed participants to use their computer to access the programme, some participants were in situations where they had to use their tablets or phones and the programme performed inconsistently. Two participants reported that it worked well, but one encountered difficulty using it on her phone as ‘*it was clunky in format*’. We requested our participants to use 2‐factor authentication for security purposes, but almost half of our participants did not activate it. For those who did, they encountered difficulties setting it up. One participant noted this step may hinder those who are older or unfamiliar with computer technology.

Theme 4 Self‐guided reflections require headspace and personal meaning.

The HOPE programme included optional self‐guided reflective checkpoints consisting of questions based on each module's content. Participants were encouraged to consider them and record their thoughts in a journal. Some participants reported that these allowed them to pause, reflect and process their feelings and emotions.

‘I liked the fact that it gave you the opportunity to […] reflect on how you were at the time’ (I)

‘It made me slow down and think about […] what am I actually feeling when these things occur and what impact does that have on me or my family’ (E)

‘…Sit with my feelings about a certain experience and then take the opportunity to write it down or just have that conversation with myself… It was interesting just to, I guess, reflect on how I found this or how I'm thinking about that’. (L)

Other participants felt that the checkpoints disrupted the flow of the programme because the reflective exercises occurred in the middle of the content.

‘*My brain just wanted to be like go, go, go, get through the questions. I didn't want to pause and reflect… My brain is not in the reflection mode*’ *(A)*. Similarly, another participant felt that reflective checkpoints should be placed at the start of the next module, so that people had more time to reflect after completing a module.

Some participants felt the questions had no personal meaning.

‘…Really challenging to do, because I don't think they necessarily applied to me’ and that some questions were too presumptive, seemingly assuming that people would ‘have a life changing light‐bulb moment’ from the content. (J)

‘…was just tired and didn't want to… I'd realised, oh, this isn't really helping me that much’. (F)

Incidental themes

Two themes unrelated to our main research aims emerged related to participants' journey with hEDS/HSD. These themes are presented in our results as they add depth and complexity to the four main themes.

Incidental theme 1: Lack of healthcare professional knowledge and therapeutic alliance.

Participants perceived a lack of therapeutic alliance between themselves and their healthcare professionals. Ten of our 12 participants recounted difficulty finding healthcare professionals familiar with their condition. Many experienced symptoms since youth and were only diagnosed as adults after finally finding the elusive hEDS/HSD‐aware healthcare professional or one who was willing to establish a therapeutic alliance. Some participants shared difficult encounters with dismissive healthcare professionals.

‘(They were) outright dismissive because it obviously presents often as neurosis or panic’. (D)

‘Why am I seeing yet another specialist who is just gonna tell me there's nothing wrong with me when there is something wrong with me? And that's the worst part, is that everyone tells you no, you're fine. All your tests are normal. But no, I'm not fine. Why am I feeling like this if I am fine?’ (C)

With the lack of hEDS/HSD awareness among the medical field, our participants had to educate themselves. Becoming more health literate became a double‐edged sword with our participants sharing encounters of how therapeutic alliances were threatened because of poor engagement from healthcare professionals.

‘(My) way of coping with EDS has been to get as much information and have had it for a very, very long time.’ (G)

‘It's a tricky one of how do you empower people without making us seem like we're hypochondriacs, who know everything, who'll bring the checklist and (say), hey, I've done the homework and this is what I've got and this is what I need, because we all know that that gets practitioners' backs up.’ (J)

Apart from arduous healthcare journeys, some participants identified the expense of searching for answers. One participant estimated spending AUD$40,000 on specialist appointments over 7 years while another said her physiotherapist was effective but expensive to go each time there was an issue. All participants reported that it took many years to find healthcare professionals who were hEDS/HSD aware and for some on disability support, it took persistence to receive the financial assistance they needed.

Some participants who found medical and/or social support from friends and family reported benefits to their health. A participant whose husband is a medical doctor, reported that it was he who first suspected she had hEDS after seeing another patient who presented similarly. Another participant found a general practitioner who had ‘*gone out of her way to learn about it (her condition) so that she can manage my treatment*’ *(C)*, and her physiotherapist was also similarly supportive and dedicated.

Incidental theme 2: Varied self‐efficacy

For people with hEDS/HSD, building or maintaining self‐efficacy is influenced by the inherent complexity and unpredictability of hEDS/HSD and the lack of medical support. Self‐efficacy can be defined as ‘an individual's belief in his or her capacity to execute behaviours necessary to produce specific performance attainments’ [30]. In our participants' context, self‐efficacy includes beliefs about their ability to control their pain, whether they can use appropriate pain management strategies, regulate their emotions and behaviour, communicate with their healthcare professionals and perform work [31]. Some of our participants appeared to have low self‐efficacy.

‘I'm pretty jaded and cynical by this point, and I think that interferes with just about everything. I'm, you know, I'm defensive and I'm anxious and I'm […] hypervigilant” and “(have) low level of life quality.’ (D)

Some other participants working as healthcare professionals reflected on how their self‐efficacy affected their work. One participant became a health practitioner because she could not find treatment/management.

‘I have ended up becoming an actual health practitioner because I couldn't get help.’ (K)

Another participant who works as a health practitioner said, ‘*It doesn't matter if I'm in pain or if I have a migraine, I'm going to work because I've got to go to work because I have, you know, 20 or 30 patients waiting to see me*.’ (J). These participants persevere with daily life and work despite their painful condition.

### 3.1 Suggestions for Improvement

Many of our interview participants saw value in hEDS/HSD research and wanted to help improve existing knowledge, care and support for themselves and others (*B*, *D*, *E*, *F* and *L*). To enhance the programme's feasibility, acceptability and appropriateness, we asked participants for suggestions to improve the programme. We organised these suggestions into two categories (Table 2): (i) strategies to improve adherence or engagement and (ii) strategies to improve content.

**Table 2:** Suggestions for improvements.

***To improve adherence or engagement***	
**Suggestions**	**Examples**
Add different graphics	Memes
Allow flexibility and longer duration	12‐months rather than 2‐months
Automated reminders	Regular progression reminders to see where each person is up to and how much more to complete
Include actionable tasks	Meditation, rest and movement breaks
Introductory material	Provide information about background to programme creators, sample snippets of the programmes to 'humanise' the programme and give idea of what programme entails
Channels for social interaction	Chat system, helplines, online support group
Pros and cons of online supports	Cons: can be negative posts that brings people down
	Pros: can swap knowledge, find recommendations for doctors
Involve other healthcare professionals	Abridged versions to send to healthcare professionals or students
Accessibility options	Mobile phone application version, video/audio format of the content for different sensory options, online version of reflective journal that can be downloaded
***To improve content***	
**Suggestions**	**Examples**
Content from other pain educators	Pain neuroscience
Introduce other types of complementary therapy	Emotional freedom technique
Topic on how to communicate the impact of EDS in social relationships	—
Topics on nutrition and weight management	—
Modification to reflective questions	Take a less assumptive tone and encourage more reflection rather than assuming someone would change their behaviour
Different levels of information	Beginner to advanced module for people to work through or to be able to begin a level they were at

## 4 Discussion

The HOPE programme was considered feasible, acceptable and appropriate for people with hEDS/HSD. The biopsychosocial approach was generally acceptable and appropriate, especially for those newer on their hEDS/HSD journey. Self‐guided reflections were acceptable and appropriate but require personal meaning and individual headspace to support participation.

Feasibility of the HOPE programme could be improved by including social interaction in the form of healthcare professional contact or online discussion forums. This desire was echoed in a previous Delphi survey, where 69% of people with hEDS/HSD felt that regular online contact with a healthcare professional as part of a condition‐specific pain management programme was important [25]. A mixed methods study from the Netherlands found similar sentiments among their participants who wanted interaction with healthcare professionals for matters that led to significant health consequences that affect their condition or treatment outcomes [32]. The desire for discussion forums or support groups also needs to be considered but several of our participants reported negative experiences that made them feel worse and as a result, disengage from support groups. Similar findings have been reported while exploring people with long term conditions and their experiences with online supports [33]. They termed this phenomenon ‘fear of negative reinforcement’, where people felt worse after interacting with those who have shared experiences. Strategies are needed to build a safe and supportive platform for people using online intervention programmes, such as making these services optional, employing condition aware healthcare professionals for virtual consultations and having moderators and clear guidelines for online discussion forums.

To improve acceptability and appropriateness of the HOPE programme, reflection questions and certain topics needed to be presented sensitively with necessary cautions. Reflection questions require appropriate timing and consideration of personal context. Pain science education done without knowledge and acknowledgement of pre‐existing medical trauma could be triggering for those who have experienced medical dismissal, and presenting the generic biology of hEDS/HSD conditions could lead to over‐medicalization. However, our research group and stakeholders, who were predominantly people with the condition, in a previous Delphi study reported that both pain science and biology education are important elements in a pain management programme [25]. Care was taken to phrase our content in a non‐suggestive manner while reinforcing that participants should always consult their trusted healthcare professional for further assistance with their condition. Online pain management programmes should be mindful of their content so that they present factual, evidence‐based information based on the context of the target condition [34]. Pain science education and self‐reflection stimuli to encourage behavioural change as part of such programmes should be delivered sensitively to consider the person's context, provide appropriate validation of their pain and encourage a healthy therapeutic alliance with their healthcare team [35]. Healthcare professionals can facilitate therapeutic alliances and the uptake of pain management programmes by continuously upskilling themselves in the hEDS/HSD condition and on how to appropriately deliver pain management through continuing professional development [36]. Organisations such as the Ehlers–Danlos Society provide online, specialist‐guided modules about hEDS/HSD and supportive tools for healthcare professionals to learn about the condition and medical management strategies [37] to help them offer high‐standard care.

Additionally, a participant's desire for tailored programmes can be fulfilled by using algorithms or artificial intelligence to allow content matching according to one's physical baseline measures, symptom experience or goals. The superiority of tailoring such programmes is mixed, with recent studies showing that effectiveness on pain‐related outcome measures was not greater compared to standardised web‐based interventions [38, 39]. There is a need for more rigorous investigation into online pain management options that are low cost, easily accessible and have the option of customisation to see if tailored interventions are more clinically effective and cost‐effective for the hEDS/HSD population.

Neurodivergence is common in our study population [40, 41, 42]. Additional needs and preferences should be considered when designing future programmes to improve feasibility, acceptability and appropriateness of the HOPE programme. User experience guidelines have been investigated and recommended for people with ADHD or autism [43, 44]. Involving neurodivergent hEDS/HSD stakeholders and website designers with understanding of neurodivergent needs could further enhance our programme. Although this requires additional costs, timing and commitment, which was not available for this pilot study, exploring opportunities to collaborate with people with hEDS/HSD to create interventional programmes combining medical knowledge, recommendations and lived experience may lead to greater success [45, 46].

### 4.1 Research and Clinical Implications

There are several recommendations based on our study results. Future improvements to the HOPE programme and related research should [1]: utilise and/or extend upon the original biopsychosocial model by considering ways to allow individualisation of content for each person. This would allow individual context and the dynamic interplay between their three domains of biopsychosocial factors to be explored [47, 48] [2]; consider targeting people newer to their diagnosis [3]; provide adaptability options for content and programme functionality such as content matching using algorithms, having different levels of information or delivering the programme using other electronic devices and [4] improve on how self‐guided reflections are delivered so that they are appropriately timed and have personal meaning. Clinically, there is a need for healthcare professionals to engage in building therapeutic alliances by upskilling in management of rarer conditions such as hEDS/HSD, increasing their relational competence by recognising the uniqueness of each person with hEDS/HSD and viewing them as experts in their own life [49]. Interpersonal skills such as kindness, active listening and being able to connect with the person seeking treatment or support are important to individuals with chronic and complex conditions, and these clinician skills help to foster the shared decision‐making relationship and improve clinical outcomes [50].

### 4.2 Limitations

There were several limitations to our study. First, although we used purposive sampling and attempted to contact a range of participants to obtain a broad perspective, those who were interviewed included mainly educated, middle‐aged women. Our study population was similar to other qualitative studies in the hEDS/HSD population [51, 52], which suggests that these groups may be more interested and have the resources to participate in research. Additionally, over half of our participants were actively or previously working in the healthcare field and can be assumed to have relatively high health literacy. Even for those not in health care, some had spent many years investigating hEDS/HSD and accumulated a wealth of knowledge related to the condition. Therefore, sampling bias is a strong possibility in our study.

A high number of programme non‐starters did not respond to our requests for interviews. We made several attempts to contact them to find out their reasons for not starting but only two responded. The reasons for people not starting are important as it helps us understand the barriers to the programme. In a study of an online positive psychology programme for EDS, 40% of incepted participants did not start the intervention [53]. Attrition rate was high with 82% not completing outcome measures post‐treatment. The authors highlighted that high attrition was common in internet‐based interventions, proposing that those who did not follow through may have been experiencing higher disease burden and challenges to completing the programme. Our small study supports this view. People with hEDS/HSD have multiple health co‐morbidities and unpredictable flareups which may impact their ability to participate in health care, so a self‐paced and accessible programme may be even more pertinent.

## 5 Conclusion

The HOPE programme appears to be feasible, acceptable and appropriate. Participant responses and suggestions for improvements will be used to improve on subsequent versions of the HOPE programme. The four major themes identified in our study provide important guidance not only for hEDS/HSD intervention programmes but has translatability value for other similar condition‐specific online management programmes. Based on the promising results of this study, enhancing the HOPE programme and conducting future large‐scale testing to further validate its effectiveness, especially for those newer to their hEDS/HSD journey, is warranted.

## Author Contributions

**Min Tze Chew:** conceptualization, investigation, writing–original draft, methodology, validation, visualization, writing–review and editing, formal analysis, project administration, data curation. **Emre Ilhan:** conceptualization, investigation, writing–original draft, methodology, validation, visualization, writing–review and editing, formal analysis, project administration, data curation, supervision. **Sarah Dennis:** investigation, writing–original draft, methodology, validation, visualization, writing–review and editing, formal analysis, project administration. **Leslie L Nicholson:** conceptualization, writing–original draft, methodology, validation, visualization, writing–review and editing, formal analysis, supervision. **Sarah Kobayashi:** conceptualization, writing–original draft, methodology, validation, visualization, writing–review and editing, formal analysis, supervision. **Cliffton Chan:** conceptualization, investigation, writing–original draft, methodology, validation, visualization, writing–review and editing, formal analysis, project administration, data curation, supervision.

## Ethics Statement

This study was approved by the Macquarie University Human Research Ethics committee (520231630154261).

## Consent

Informed written (obtained online via Macquarie University's RedCAP platform) and verbal consent was sought from participants before their interview.

## Conflicts of Interest

The authors declare no conflicts of interest.

## Supporting information

Supporting information.

## Data Availability

Interview transcripts are available upon request as they contain health related and possible identifiers which constitutes sensitive information. Anyone wanting access to the data should contact the data custodian Cliffton Chan (cliffton.chan@mq.edu.au). For more information, please visit https://doi.org/10.25949/28225172.v1.
