# Supplementary material for: Navigating HOPE (Hypermobile Online Pain managemEnt): Perspectives and Experiences From People With Hypermobile Ehlers–Danlos Syndrome or Hypermobility Spectrum Disorder on a Condition‐Specific Online Pain Management Programme
Source: Health Expect. 2025 Feb 17;28(1):e70186. doi: 10.1111/hex.70186 (PMC11832430; doi:10.1111/hex.70186)
Supplement: Supplementary file 1 — Supporting information. [file HEX-28-e70186-s001.pdf]

Supplement A – Interview questions

1. Briefly, can you tell us why you decided to take part in this study?
2. What do you think about the features of the website? (features such as readability of the font, the use of images, additional documents)
3. What are your thoughts about the content of the website?
4. What are your thoughts on the usefulness of the HOPE program?
5. How has the program made a difference to your pain?
6. Do you recall that in some of the modules, there were checkpoints. These checkpoints included mini quizzes and reflection questions about your own pain journey and experience. What are your impressions about those checkpoints?
7. Thinking just about the program, what helped you stick to the program? What suggestions would you have for others to help them complete this program? (for completers) OR What strategies or things we could have implemented that would have helped you complete the program? (for non-completers)
8. Thinking beyond just the program, was there anything that facilitated/encouraged you to complete the program? (for completers) OR was there anything that hindered/discouraged you from completing the program? (for non-completers)
9. Was there anything else that we have not mentioned, that made it difficult to engage with the program? After finishing (completers) / seeing some elements of the HOPE program (non-completers), do you think you will go about managing your pain differently or the same in the future? Why do you say that?
10. Are there any changes you would suggest to improve the program, in terms of both the content and website? What makes you say that?
